# Long-Term Trend in the Association Between Disaster Damage and Happiness Before and After the Great East Japan Earthquake

**DOI:** 10.3389/ijph.2022.1604901

**Published:** 2022-09-14

**Authors:** Masato Nagai, Hiroyuki Hikichi, Koichiro Shiba, Katsunori Kondo, Ichiro Kawachi, Jun Aida

**Affiliations:** ^1^ Department of Oral Health Promotion, Graduate School of Medical and Dental Sciences, Tokyo Medical and Dental University, Tokyo, Japan; ^2^ Division of Public Health, Kitasato University School of Medicine, Sagamihara, Japan; ^3^ Department of Social and Behavioral Sciences, Harvard T.H. Chan School of Public Health, Boston, MA, United States; ^4^ Department of Epidemiology, Harvard T.H. Chan School of Public Health, Boston, MA, United States; ^5^ Department of Social Preventive Medical Sciences, Center for Preventive Medical Sciences, Chiba University, Chiba, Japan; ^6^ Department of Gerontological Evaluation, Center for Gerontology and Social Science, National Center for Geriatrics and Gerontology, Obu, Japan

**Keywords:** disaster, trend, happiness, housing damage, older people

## Abstract

**Objectives:** Disasters change survivors’ living circumstances, which can affect their happiness. We examined the trends in the association between disaster damage and happiness before and after a disaster.

**Methods:** We analyzed 4,044 participants aged ≥65 years who had experienced the Great East Japan Earthquake in 2011. The baseline survey was conducted 7 months before the disaster. Follow-up surveys have been conducted every 3 years. Using a mixed model for repeated measures, we compared the prevalence ratios (PRs) for unhappiness according to the survivors’ level of housing damage, which is a proxy for disaster damage.

**Results:** The unhappiness in participants who suffered severe damage appeared to be higher than in those with no damage in 2010 (multivariate-adjusted PR: 1.18, 95% confidence interval: 0.93–1.48). A higher PR was observed after the earthquake in 2013 (1.34, 0.79–2.28), while there was no difference in 2016 (1.02, 0.53–1.97) and 2019 (1.03, 0.50–2.12).

**Conclusion:** The prevalence of unhappiness in survivors with severe housing damage was higher before the disaster. However, the unhappiness gap between people with and without housing damage converged during the follow-up.

## Introduction

Happiness is a component of well-being and refers to positive emotions that reflect an individual’s life satisfaction [1, 2]. While happiness itself is a highly valued outcome [3], it is also associated with health outcomes such as mental health [2, 4–6] and suicide [7–9] as well as health behaviors such as drinking [10, 11], healthy dietary habits [11–15], smoking [10, 11, 15], and physical activity [10–22]. Some of these associations are likely to be due to reverse causation, that is, poor sleep causing unhappiness.

On 11 March 2011, the Great East Japan Earthquake, which registered a magnitude of 9.0 on the Richter scale, struck the northeast Pacific coast of northern Japan and caused a tsunami and nuclear plant meltdown. This devastating disaster affected the lives of 18,000 people and caused widespread property destruction [23]. Beyond the immediate loss of lives and damage, the major disaster also caused changes in the survivors’ living circumstances, which could potentially influence happiness in the long-term period. For example, numerous victims were forced to relocate following home loss or radiation leakage.

This study aims to examine the long-term trajectory of happiness among disaster survivors from 2010 to 2019, as little is known regarding this subject. Hypothetically, it is possible that survivors may find their reference points shifting as a result of suffering major damage (e.g., property loss), thereby forcing them to rethink their life goals and values. That is, in the immediate aftermath of a disaster, people may experience increased unhappiness, but their goals and expectations may get adjusted over time, leading them to ultimately return to a level of happiness comparable to pre-disaster levels. On the other hand, some survivors may experience prolonged unhappiness due to ongoing difficulties, such as health problems and financial insecurity.

Additionally, previous studies on the association between disaster damage and health outcomes among disaster victims have generally collected data only after the disaster. There is a possibility that there is a pre-existing difference in health outcomes between damage situations before the disaster. Hence, it is important to understand how much of the unhappiness can be attributed to traumatic disaster experiences pre-dating the event. A unique strength of our study design is that the measurement of happiness was conducted 7 months prior to the occurrence of the earthquake and tsunami.

## Methods

### Study Participants

A longitudinal study was conducted using repeatedly measured data from the Iwanuma Study, which is part of a larger nationwide cohort study conducted, called the JAGES (Japan Gerontological Evaluation Study) [24], which was established in 2010. The JAGES is a large-scale prospective cohort study that is designed to describe the health status and social determinants of health in people aged 65 years or over without long-term care needs. Iwanuma is located approximately 80 km west of the epicenter of the 2011 earthquake. At baseline, a census was undertaken of all residents of Iwanuma aged 65 years or older.

Surveys were mailed in August 2010, 7 months before the earthquake. Of the 8,576 eligible residents of Iwanuma, 5,058 responded (response rate: 59.0%). After the earthquake and tsunami, we followed up with 4,957 respondents, excluding those with invalid IDs or lacking sex/age information (*n* = 101). Follow-up surveys have been conducted with these participants every 3 years *via* a door-to-door survey that excluded participants who died or moved away from Iwanuma during the follow-up period.

**FIGURE 1:**
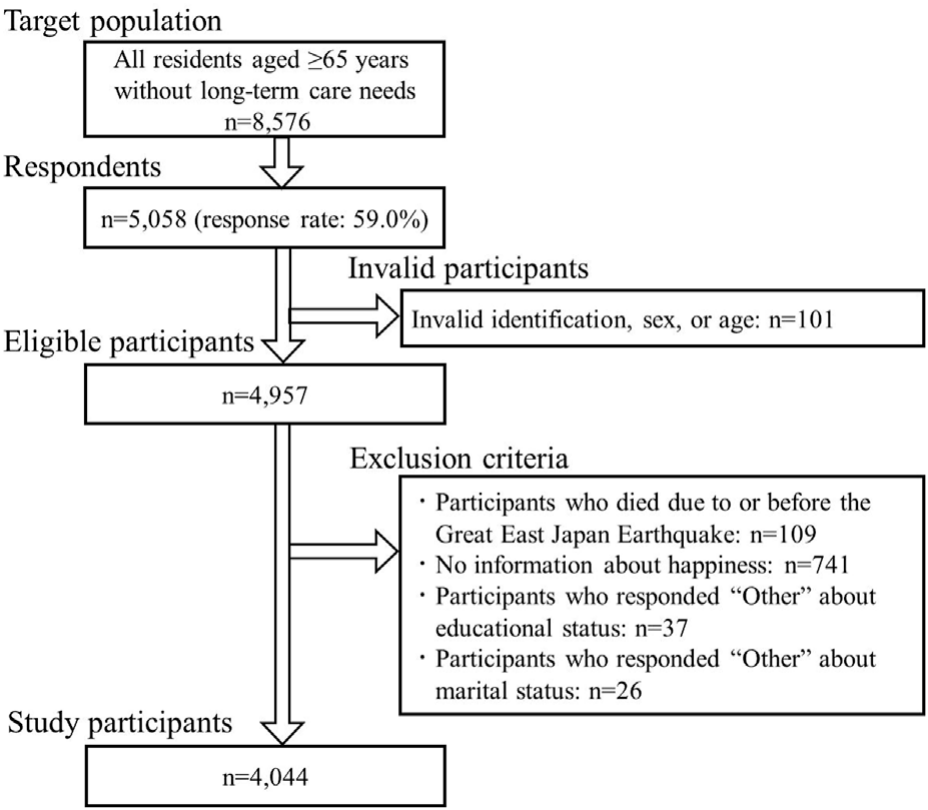
Flow diagram of the study participants (Iwanuma, Japan, 2010).

For the current analysis, we excluded 109 participants who died before or on the day of the earthquake. In addition, 741 participants with missing information about happiness, 37 participants who responded “Other” to educational status, and 26 participants who responded “Other” to marital status in the 2010 survey were excluded. Consequently, 4,044 participants aged ≥65 years were included in the analysis (Figure 1).

The survey protocol was reviewed and approved by the Ethics Committees on Research of Human Subjects at the Harvard T.H. Chan School of Public Health (CR-23143), Tohoku University (21-40, 24-29), Nihon Fukushi University (10-05, 13-14), and Chiba University (2493). Informed consent was obtained at the time of data collection at Nihon Fukushi University (Approval No. 10-05). Written informed consent was obtained upon the voluntary return of the questionnaire.

### Disaster Damage

In this study, we considered housing damage as a proxy for comprehensive disaster damage. Damage from the Great East Japan Earthquake was mainly caused by the tsunami, and residential houses were damaged by it as well. Housing damage affects an individual’s life in the long-term, such as through changes in the living environment and new mortgages.

In 2013, participants were asked a question about the level of housing damage based on an objective assessment by two or more technical officers inspecting each residential property. The level of housing damage was based on the official designations determined by the local government for compensation. Based on a previous study [25], we categorized the participants into the following three categories: “no damage,” “half/partly destroyed” (response: “Half destroyed” or “Partially destroyed”), or “totally/mostly destroyed” (response: “Total collapse” or “Partial but extensive collapse”).

### Happiness

Happiness in 2010, 2013, 2016, and 2019 was assessed with the following question, which is part of the Geriatric Depression Scale (GDS) [26, 27]: “Do you feel happy most of the time?”. The participants were asked to choose “yes” or “no”; those who responded “no” were defined as “unhappy”.

### Confounders

We used the following variables from the 2010 baseline survey as confounders: sex (men or women), age (65–69 years, 70–74 years, 75–79 years, 80–84 years, or ≥85 years), living alone (yes or no), history of cardiovascular disease (CVD) or cancer (yes or no), smoking (never smoked, quit, or current smoker), drinking (non-drinker, quit, or current drinker), marital status (yes or no), and depression (yes or no). Depression was evaluated using the GDS [26, 27], and the results were classified as “no depression” (<5 points), or “depressive symptoms” (≥5 points) [28, 29]. We also controlled for social cohesion, social participation, and reciprocity [30]. Social cohesion was assessed using three questions: 1) “Do you think that people living in your community can be trusted in general?” (Generalized trust); 2) “Do you think people living in your community try to help others in most situations?” (Mutual help); and 3) “How attached are you to the community in which you live?” (Community attachment). Responses were measured on a five-point Likert scale (1 Very, 2 Moderately, 3 Neutral, 4 Not really, or 5 Not at all). Similarly, social participation was evaluated using three questions: 1) “How often do you attend sports club activities?”; 2) “How often do you attend hobby groups?”; and 3) “How often do you attend volunteer groups?”. Responses were measured as frequencies (1 ≥ 4 times/week, 2 2–3 times/week, 3 1 time/week, 4 1–3 times/month, 5 A few times/year, or 6 Never). Reciprocity was evaluated using three questions: 1) “Do you listen to someone’s concerns and complaints?” (Providing emotional support); 2) “Do you have someone who listens to your concerns and complaints?” (Receiving emotional support); and 3) “Do you have someone who looks after you when you are sick for a few days?” (Receiving instrumental support). The possible responses were “yes” or “no”.

In model 2, we additionally adjusted for socioeconomic status (SES), that is, education level (<10 years, 10–12 years, or ≥13 years), current working (yes or no), and equivalent income (quartile).

### Statistical Analysis

A mixed model for repeated measures by Poisson distribution with robust error variance was used to derive prevalence ratios (PRs) and 95% confidence intervals (CIs) for unhappiness [31]. “No damage” was set as the reference. We included survey years and its product term by level of housing damage in the model. The correlation between repeated outcome measurements for the same participants across different survey years was accounted for by including random effects for the individuals. In the multivariate-adjusted model, we controlled for all pre-disaster characteristics as potential confounders. Missing information regarding the level of housing damage and covariates was imputed via multiple imputation by fully conditional specification, in which we combined estimates from 20 imputed datasets [32]. All *p* values were two-tailed, and differences of <0.05 were considered as statistically significant. We used the SAS version 9.4 statistical software package.

## Results

### Baseline Characteristics by Level of Housing Damage

Table 1 shows the characteristics of the study participants according to the categories of housing damage for men and women. More severe housing damage was associated with a higher prevalence of unhappiness in 2010, 7 months before the earthquake (e.g., prevalence = 11.9% for no damage versus 17.5% for totally/mostly destroyed). The baseline difference in unhappiness decreased during the follow-up period.

**TABLE 1:** Characteristics by the level of housing damage among 4,044 men and women aged ≥65 years (Iwanuma, Japan, 2010).

	No damage	Half/partly destroyed	Totally/mostly destroyed	Missing
No. of participants	1,283	1,577	246	938
Unhappiness in 2010 (%)	11.9	13.3	17.5	16.7
Unhappiness in 2013 (%)	10.8	11.9	17.9	2.4
Missing	1.6	1.0	2.0	91.5
Unhappiness in 2016 (%)	6.6	6.7	8.1	4.1
Missing	36.5	33.4	40.7	68.4
Unhappiness in 2019 (%)	5.6	5.5	6.9	3.3
Missing	46.1	45.2	51.6	72.5
Men (%)	47.0	44.6	41.5	47.6
Age (%)				
65–69 years	30.5	31.9	32.1	24.8
70–74 years	31.2	28.7	27.2	25.4
75–79 years	20.8	23.5	21.5	21.9
80–84 years	12.0	11.0	11.4	17.2
≥85 years	5.5	4.9	7.7	10.8
Living alone (%)	9.8	7.6	4.9	10.0
Missing	0.9	1.2	3.3	3.3
History of CVD^a^ or cancer (%)	18.2	18.2	22.8	24.2
Missing	25.6	23.4	23.2	23.2
Smoking (%)				
Current smoker	10.5	10.0	13.0	12.6
Quit	28.1	25.8	20.7	27.5
Never smoked	55.0	56.8	55.3	50.9
Missing	6.3	7.5	11.0	9.1
Drinking (%)				
Current drinker	38.2	38.9	29.3	34.3
Quit	3.3	2.9	5.3	4.4
Non-drinker	57.1	56.4	61.0	59.3
Missing	1.4	1.8	4.5	2.0
Married (%)	72.1	74.6	67.5	65.5
Missing	1.6	1.5	5.3	3.3
Depression (%)	24.2	27.3	29.3	31.5
Missing	9.7	10.2	13.0	13.7
Generalized trust (%)				
Very	10.6	13.8	15.9	12.4
Moderately	58.1	55.1	51.2	50.1
Neutral	24.6	25.6	20.7	28.0
Slightly or not at all	5.1	4.5	8.5	6.8
Missing	1.6	1.0	3.7	2.7
Mutual help (%)				
Very	5.9	8.1	11.0	9.1
Moderately	50.9	50.7	44.3	43.1
Neutral	32.0	30.2	28.1	32.8
Slightly or not at all	9.0	9.2	12.2	10.9
Missing	2.3	1.8	4.5	4.2
Community attachment (%)				
Very	23.4	27.9	37.0	24.7
Moderately	55.2	52.8	41.1	45.2
Neutral	14.2	14.1	13.0	18.7
Slightly or not at all	5.9	4.4	6.5	9.3
Missing	1.3	0.7	2.4	2.1
Sports club activities (%)				
≥1 time/week	16.8	19.5	7.3	9.9
1–2 times/month	5.1	5.9	3.7	4.5
A few times/year	4.4	6.1	5.3	3.4
Never	59.9	55.2	58.5	63.3
Missing	13.7	13.3	25.2	18.9
Hobby groups (%)				
≥1 time/week	19.3	23.7	10.2	14.8
1–2 times/month	16.0	16.7	11.4	10.3
A few times/year	9.1	9.3	10.2	6.2
Never	43.7	38.4	43.9	51.4
Missing	12.0	11.9	24.4	17.3
Volunteer groups (%)				
≥1 time/week	3.3	4.0	2.0	3.3
1–2 times/month	6.2	7.3	4.5	3.4
A few times/year	7.1	9.1	11.0	4.7
Never	64.5	62.1	54.1	65.5
Missing	18.9	17.5	28.5	23.1
Providing emotional support (%)	90.6	91.9	89.8	86.8
Missing	2.8	3.4	4.9	5.1
Receiving emotional support (%)	89.7	91.4	87.4	82.6
Missing	3.6	3.2	6.5	6.9
Receiving instrumental support (%)	93.4	95.8	93.5	90.4
Missing	1.8	1.8	3.3	3.8
Education years (%)				
<10 years	31.3	32.7	58.9	39.8
10–12 years	46.1	43.8	26.4	40.3
≥13 years	21.4	22.2	12.2	17.4
Missing	1.3	1.3	2.4	2.6
Current working (%)	14.1	18.5	19.5	13.4
Missing	9.2	9.8	16.7	15.1
Equivalent income (%)				
Q1	20.1	19.0	32.9	24.0
Q2	20.7	20.1	17.5	19.1
Q3	22.5	23.5	15.0	18.0
Q4	22.1	21.4	13.0	18.3
Missing	14.5	16.0	21.5	20.6

a CVD, cardiovascular disease.

In participants with totally/mostly destroyed homes, the prevalence of shorter education years, current working, lower equivalent income, history of CVD or cancer, current smoker, depression, poor social cohesion, poor social participation, and poor reciprocity was the highest compared with the other participants. Meanwhile, the prevalence of current drinker and married was the lowest.

### The Trends in the Association Between Housing Damage and Unhappiness

Table 2 shows the association between housing damage and the prevalence of unhappiness in 2010, 2013, 2016, and 2019. After adjusting for pre-disaster characteristics, the point estimates of multivariate-adjusted PR1 in the totally/mostly destroyed homes (versus no damage) increased in 2010 (PR: 1.25, 95% CIs: 0.99–1.57). A similar finding was observed in 2013 (1.42, 0.84–2.42). Meanwhile, in 2016 and 2019, the prevalence of unhappiness decreased in participants with totally/mostly destroyed homes and no obvious difference was observed between them and those with no damage in 2016 (1.07, 0.56–2.08) and 2019 (1.08, 0.53–2.23). After further adjustment for SES, such as education level, current working, and equivalent income, the results of multivariate-adjusted PR2 showed a similar tendency to multivariate-adjusted PR1, but point estimates were slightly attenuated.

**TABLE 2:** PRs^a^ and 95% CIs^a^ of unhappiness according to the level of housing damage (Iwanuma, Japan, 2010, 2013, 2016, and 2019).

	No damage	Half/partly destroyed	Totally/mostly destroyed
No. of participants	1,660	2,033	351
Unhappiness in 2010 (%)			
Crude PRs (95% CIs)	Reference	1.11 (0.94–1.31)	1.51 (1.18–1.94)
Sex-age-adjusted PRs (95% CIs)	Reference	1.11 (0.94–1.31)	1.54 (1.20–1.98)
Multivariate-adjusted PRs1^b^ (95% CIs)	Reference	1.08 (0.90–1.28)	1.25 (0.99–1.57)
Multivariate-adjusted PRs2^c^ (95% CIs)	Reference	1.09 (0.91–1.29)	1.18 (0.93–1.48)
Unhappiness in 2013 (%)			
Crude PRs (95% CIs)	Reference	1.05 (0.73–1.51)	1.65 (0.97–2.79)
Sex-age-adjusted PRs (95% CIs)	Reference	1.05 (0.73–1.51)	1.67 (0.98–2.84)
Multivariate-adjusted PRs1 (95% CIs)	Reference	1.06 (0.72–1.58)	1.42 (0.84–2.42)
Multivariate-adjusted PRs2 (95% CIs)	Reference	1.07 (0.73–1.58)	1.34 (0.79–2.28)
Unhappiness in 2016 (%)			
Crude PRs (95% CIs)	Reference	0.90 (0.60–1.35)	1.23 (0.64–2.35)
Sex-age-adjusted PRs (95% CIs)	Reference	0.89 (0.59–1.34)	1.23 (0.64–2.39)
Multivariate-adjusted PRs1 (95% CIs)	Reference	0.97 (0.63–1.51)	1.07 (0.56–2.08)
Multivariate-adjusted PRs2 (95% CIs)	Reference	0.98 (0.64–1.52)	1.02 (0.53–1.97)
Unhappiness in 2019 (%)			
Crude PRs (95% CIs)	Reference	0.93 (0.60–1.43)	1.14 (0.56–2.34)
Sex-age-adjusted PRs (95% CIs)	Reference	0.92 (0.59–1.44)	1.17 (0.56–2.44)
Multivariate-adjusted PRs1 (95% CIs)	Reference	0.94 (0.59–1.49)	1.08 (0.53–2.23)
Multivariate-adjusted PRs2 (95% CIs)	Reference	0.95 (0.60–1.51)	1.03 (0.50–2.12)

a PR, prevalence ratio; CI, confidence interval.

b Multivariate-adjusted PRs1 were adjusted for sex (men or women), age (65–69 years, 70–74 years, 75–79 years, 80–84 years, or ≥85 years), living alone (yes or no), history of cardiovascular disease or cancer (yes or no), smoking (never smoked, quit, or current smoker), drinking (non-drinker, quit, or current drinker), marital status (yes or no), depression (yes or no), generalized trust (very, moderately, neutral, or slightly or not at all), mutual help (very, moderately, neutral, or slightly or not at all), community attachment (very, moderately, neutral, or slightly or not at all), sports club activities (≥1 time/week, 1–2 times/month, a few times/year, or never), hobby groups (≥1 time/week, 1–2 times/month, a few times/year, or never), volunteer groups (≥1 time/week, 1–2 times/month, a few times/year, or never), providing emotional support (yes or no), and receiving emotional support (yes or no), and receiving instrumental support (yes or no).

c Multivariate-adjusted PRs2 were adjusted for variables in multivariate-adjusted PRs1 plus education level (<10 years, 10–12 years, or ≥13 years), current working (yes or no), and equivalent income (quartile).

**FIGURE 2:**
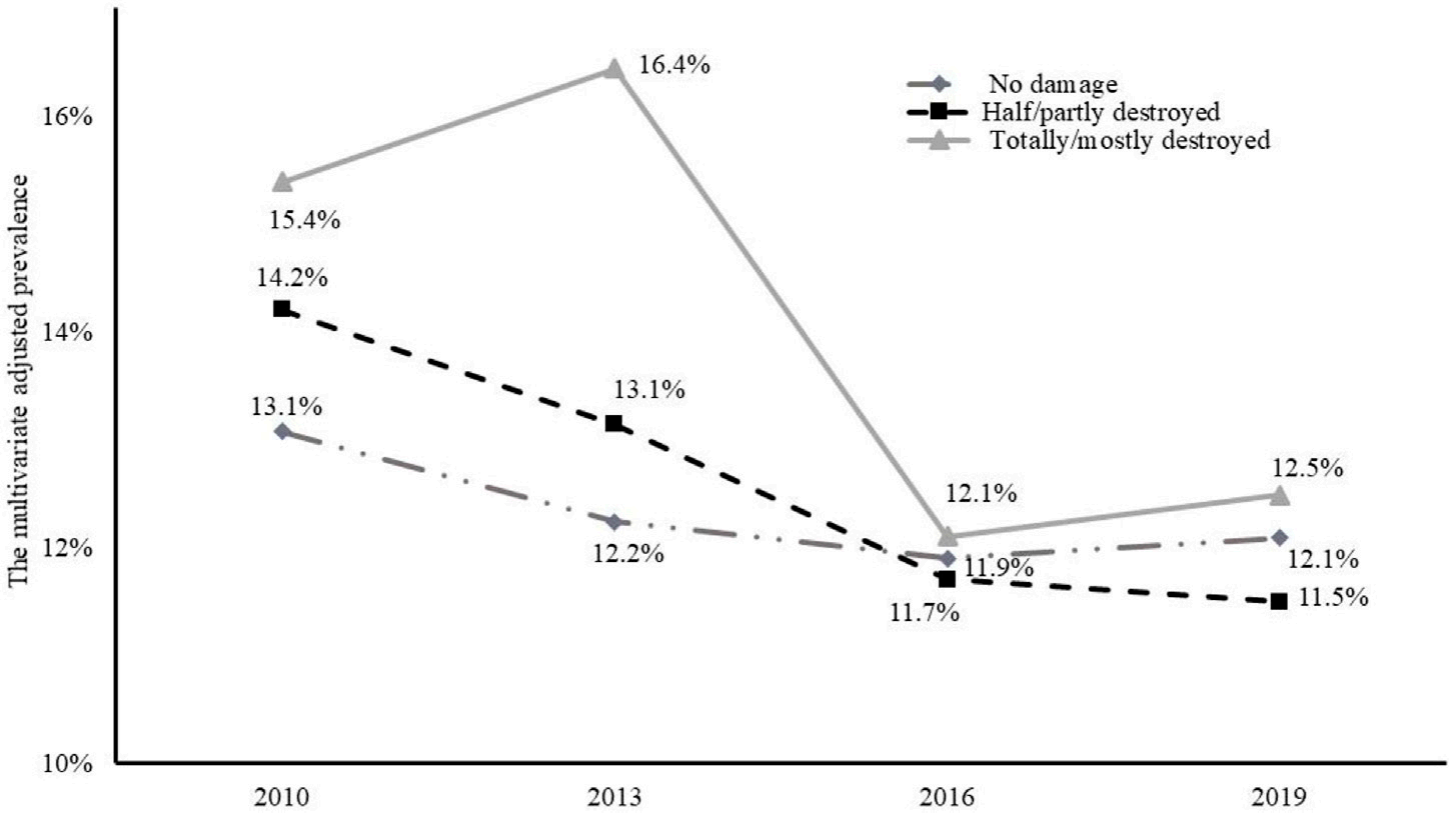
The multivariate-adjusted prevalence of unhappiness according to the level of housing damage in each year (Iwanuma, Japan, 2010, 2013, 2016, and 2019). Multivariate-adjusted prevalence was estimated using a mixed model for repeated measures by Poisson distribution with robust error variance adjusted for confounders.

Figure 2 illustrates the prevalence of unhappiness for each housing damage category and year predicted from the multivariate-adjusted mixed model for repeated measures. The prevalence of unhappiness tended to decrease over time in all categories of housing damage, but this trend was more obvious in the totally/mostly destroyed category.

## Discussion

This study examined trends in the association between disaster damage and unhappiness after the earthquake from the viewpoint of the level of housing damage. We found that the prevalence of unhappiness might be higher in participants with the most severe housing damage than in those with no damage, especially immediately after the earthquake. However, this difference progressively diminished during the follow-up.

Irrespective of the level of housing damage, the prevalence of unhappiness tends to decrease over time. Several studies have shown that feelings of happiness have a U-shaped association with age throughout the life course [33–35], which suggests that older adults tend to pay more attention to positive information [36, 37]. The decreasing trend in the present results was caused by aging during the follow-up.

The study participants received several forms of public support, such as free medical care, tax allowance, relief money, and community formation. In addition, people often rate their present level of happiness compared to the past; thus, the disaster may have lowered the reference point for happiness, and survivors may have adjusted to their current circumstances. In particular, survivors who had experienced housing loss were initially moved to temporary trailer homes and lived in cramped conditions for approximately 5 years. By 2016, they were finally moved to permanent housing, which likely contributed to improving their level of happiness. These life experiences may have caused changes in their lifestyles and sense of value, making it easier for them to perceive their current situation as happy. According to the theory of hedonic adaptation, both favorable events (e.g., winning the lottery) and unfavorable events (e.g., being paralyzed) result in long-run adaptation, so that people return to their happiness baseline over time [38]. Our findings are consistent with the concept of hedonic adaptation, i.e., over an 8-year follow-up after the disaster, the unhappiness levels of people who suffered housing damage tended to converge with the levels of those who had escaped damage.

The earthquake served as a natural experiment. Participants were randomly exposed; however, the prevalence of unhappiness might differ between levels of housing damage before the earthquake. During the earthquake, housing damage was caused by the tsunami rather than by the tremors or fire. The magnitude of housing damage was worse in regions close to the coastline, underscoring the influence of SES on where people lived. In fact, SES characteristics, such as education level, current working, and equivalent income before the earthquake, differed between levels of housing damage. Hence, the difference in the prevalence of unhappiness between levels of housing damage before the earthquake is due to the regional specifics. PRs were attenuated after adjusting for baseline factors, including SES, before the disaster. These differences might disappear if unmeasured SES is adjusted for as well.

Several studies have shown that feelings of happiness are associated with healthy behaviors [10–15, 17–22, 39–42]. After a disaster, victims tend to experience deteriorated health [43–47] and engage in unhealthy behaviors [48–51], though some of these conditions (e.g., depression) can be overcome in the long-term [45, 47]. Satisfying subjective happiness in victims might reduce unhealthy behaviors and contribute to the recovery of their health. A major strength of the present study is that it is the first to examine the association between housing damage and happiness. Additionally, in studies on disasters, data are usually collected after the disaster; however, the baseline data of this study were collected just before the earthquake. Thus, we can discuss the effects of the disaster by considering the status before the disaster without the possibility of recall bias. In the present results, it can be interpreted that the higher prevalence of unhappiness in 2013 in the group with the most severe housing damage was not caused by the earthquake, and the difference might have existed before the earthquake. Information about disaster damage is usually affected by survival and response bias, as only survivors can respond to a questionnaire, and those with severe damage tend to be non-responsive and lost to follow-up [52]. For this reason, studies on disasters tend to underestimate their effects; however, the present study reduced this bias through multiple imputation and a mixed model for repeated measures.

Nevertheless, this study has several limitations. First, as discussed above, the results might be residually biased because of unmeasured confounders, such as wealth. We acknowledge that the reason for the pre-disaster difference might be the presence of other characteristics associated with geographic regions (and, hence, housing damage) and happiness. However, proximity to the coastline (which approximates the geographic location of a household) was strongly correlated with housing damage. Adjusting for information about location in the analysis would cause a serious multicollinearity problem. Secondly, the results should be interpreted with caution due to the high prevalence of missing information about happiness during the follow-up period. However, with the use of a mixed model for repeated measures, an unbiased estimator for missing data can be obtained if the pattern of missing data is missing completely at random or missing at random (MAR) [53, 54]. Meanwhile, we referred to unmeasured variables elsewhere. In this case, the MAR assumption cannot be satisfied, so the possibility of bias cannot be ruled out. Third, unhappiness was assessed using only a single question with binary options, thus affecting reliability. Some of the participants were asked the following question: “To what degree do you feel you are currently happy?” (Score “0” for “Very unhappy” and “10” for “Very happy”). We created a cross-tabulation between the present outcome and this question (Supplementary Table S1). There seems to be a cutoff between points four and five, and the present outcome has a certain validity (Spearman’s rank correlation coefficient; *r* = 0.351, *p* < 0.001). Fourth, our first follow-up survey, including a question about happiness, was conducted almost 2.5 years after the earthquake. However, although the association between the level of housing damage and happiness immediately after the disaster remains unknown, the aim of the present study was to examine the long-term trends in this association, including those before the disaster.

In conclusion, the present study showed that the long-term trend in the prevalence of unhappiness in victims of severe housing damage due to the earthquake decreased until 2019. Because feelings of happiness are associated with healthy behaviors, it might be helpful to recover the health status of disaster victims.
