# Supplementary material for: Long-Term Trend in the Association Between Disaster Damage and Happiness Before and After the Great East Japan Earthquake
Source: Int J Public Health. 2022 Sep 14;67:1604901. doi: 10.3389/ijph.2022.1604901 (PMC9515324; doi:10.3389/ijph.2022.1604901)
Supplement: Supplementary file 1 [file Table1.DOCX]

| Supplemental Table 1. Participants’ answers to two questions about happiness (Iwanuma, Japan, 2010, 2013, 2016, and 2019) | | | | | | | | | | | | |
| --- | --- | --- | --- | --- | --- | --- | --- | --- | --- | --- | --- | --- |
|  | To what degree do you feel you are currently happy? (Score “0” for “Very unhappy” and “10” for “Very happy”) | | | | | | | | | | | |
|  | 0 | 1 | 2 | 3 | 4 | 5 | 6 | 7 | 8 | 9 | 10 | Total |
| Do you feel happy most of the time: Yes | 7 | 7 | 11 | 53 | 78 | 1,091 | 855 | 1,372 | 1,979 | 794 | 1,031 | 7,228 |
| Do you feel happy most of the time: No | 11 | 45 | 31 | 90 | 94 | 352 | 122 | 103 | 53 | 20 | 28 | 949 |
